# Do vaginal microbicides reduce the risk of HIV acquisition in women?

**DOI:** 10.11604/pamj.2022.43.96.30227

**Published:** 2022-10-24

**Authors:** Jael Apondi Obiero, Paul Ogongo, Peter Gichuhi Mwethera, Charles Shey Wiysonge

**Affiliations:** 1 Department of Reproductive Health & Biology, Institute of Primate Research, Nairobi, Kenya,; 2 Cochrane Kenya, Kenya Medical Research Institute, Nairobi, Kenya,; 3 Department of Tropical and Infectious Diseases, Institute of Primate Research, Nairobi, Kenya,; 4 Division of Experimental Medicine, Department of Medicine, University of California, San Francisco, San Francisco, CA, USA,; 5 Cochrane South Africa, South African Medical Research Council, Cape Town, South Africa,; 6 School of Public Health and Family Medicine, University of Cape Town, Cape Town, South Africa,; 7 Department of Global Health, Stellenbosch University, Cape Town, South Africa,; 8 HIV and other Infectious Diseases Research Unit South African Medical Research Council, Durban, South Africa

**Keywords:** Microbicides, HIV, women

## Abstract

Currently, no single HIV prevention method meets the needs of all people at risk of infection and a range of options are needed for individuals to protect themselves and to curb the HIV epidemic. Many people living with HIV or at risk for HIV infection in low and middle-income countries do not have access to prevention, treatment and care, and there is still no cure. Despite large preventive efforts, HIV acquisition rates remain unacceptably high and transmission mainly occurs through heterosexual intercourse, where women are significantly more vulnerable to infection than men. Widespread violence, many sociocultural and economic factors in these regions limit the ability of women to insist on safer sexual practices to decrease HIV transmission risks. The development of vaginal HIV microbicides, the use of which would be discretely controlled or initiated by women, has therefore attracted much interest as a strategy to help prevent HIV sexual transmission. In this commentary, we discuss the evolution of vaginal microbicies, the different types that have undergone clinical trials, the past challenges to future hopes, the products that are currently in use and implications for women who are at risk to HIV infection in sub-Saharan Africa.

## Commentary

Globally, there have been advances in the scientific understanding of HIV, its prevention and treatment, as well as years of significant effort by the global health community and leading government and civil society organizations. Due to the numerous antiretroviral (ART) drugs with different mechanisms of action currently available for treatment of HIV, the goal of ending the AIDS epidemic has become attainable through elimination of HIV and AIDS-related deaths in most parts of the world. In the past decade, new global efforts have seen the development and scaling up of highly effective and safe prevention tools, most notably using ART medications as both pre-exposure prophylaxis (PrEP) by uninfected persons to prevent HIV acquisition and as treatment for persons living with HIV to eliminate infectiousness [1]. Both antiretroviral PrEP and treatment supplement the longer-standing HIV prevention strategies including testing, condom use, behavioral risk reduction, voluntary medical male circumcision, harm reduction interventions for persons who inject drugs, and treatment and prevention of other sexually transmitted infections (STIs).

Despite this progress, many people living with HIV or at risk of HIV acquisition, particularly in low and middle-income countries (LMICs), still do not have access to prevention, care, and treatment. Adolescent girls and young women aged 15-24 account for approximately 5000 new infections each week globally, with most in sub-Saharan Africa (SSA). SSA is home to two-thirds (67%) of people living with HIV and women in some African settings continue to bear a disproportionate burden of the global HIV-1 epidemic. Recent evidence has demonstrated that HIV incidence in young African women in some settings has seen no substantial decline over the past decade. Other than biological factors, the high risk of HIV infection among adolescent girls and young women in these regions is further amplified by widespread sexual violence, stigma, discrimination, poverty and gender equity factors. Furthermore, it is important to note that many LMICs hardest hit by HIV also face serious challenges due to other infectious diseases, food insecurity, additional global health and development problems thus hindering HIV prevention access [2]. For example, the recent global spread of COVID-19 has been reported to have detrimental effects on HIV/AIDS response in LMICs including disruptions in access to AR medicines and preventive services. Such interruptions could result in many more new infections and HIV- related deaths [3]. Thus, new prevention options, particularly ones that can be delivered at scale and to populations not currently benefiting from prevention access, are needed. This necessitated the drive for the study of vaginal microbicides, acceptable woman-initiated methods to prevent sexual transmission of HIV from men to women.

The development of vaginal HIV microbicides, self-administered prophylactic agents applied to the vagina, the use of which would be controlled by women as a strategy to prevent new HIV sexual transmission infections emerged nearly three decades ago. The successful development of these agents for the prevention of HIV infection requires significant levels of protection against HIV acquisition, demonstrated safety in healthy populations, and no adverse interactions with commonly used vaginal drugs or other HIV prevention approaches. The initial trend in the pursuit of an effective microbicide involved the use of non-HIV-specific substances to prevent the entry of the virus in order to block the first step of infection. For nearly two decades, the first-generation microbicides tested for efficacy, mostly in SSA, were formulated as gels without direct antiviral activity against HIV and included detergents; nonoxynol-9 (N-9) and C31G 9 (SAVVY), polyanions; Carraguard, PRO 2000 and cellulose sulphate (CS) and a vaginal acidifier BufferGel. While initially the early-phase clinical studies of these microbicides were promising, the results of larger trials failed to demonstrate efficacy [4]. Suggested reasons for failure include alteration of the vaginal microbiota on application that yielded a vaginal environment that lost its natural protective abilities, either directly enhancing HIV transmission or indirectly acting to activate potential host cells, which would facilitate HIV transmission [2].

After unsuccessfully evaluating numerous compounds in clinical trials, the focus has switched to the study of potential microbicides with antiretroviral drugs that prevent virus replication. The first antiretroviral product evaluated was a gel containing 1% tenofovir (TNF), a nucleoside reverse-transcriptase inhibitor (NRTI) which acts in the case of HIV infection by blocking the activity of reverse transcriptase and preventing the virus from infecting cells and replicating. In 2010, a proof-of-concept for human HIV prevention by antiretroviral-based vaginal microbicides (ARV-VMB) was achieved in one clinical trial, the CAPRISA 004 study, in South African women. The 1% TNF gel was found to reduce HIV infection rate by 39% in women using the gel up to 12 hours before sex and again within 12 hours after sex, compared with women using a placebo gel [5]. Partial protection in this study was mainly attributed to poor adherence which could have accounted for low or fluctuating levels of TNF throughout the timeframe of potential viral exposure leading to insufficient efficacy. Despite this first successful clinical trial, subsequent trials of this product failed to confirm the potential for protection, in large part due to low adherence and were stopped prematurely. Other factors that influenced the effectiveness of the TNF gel are reported to include adhesion of the formulation to the mucosa which could alter drug concentration; differential drug metabolism by vaginal microbiota, adherence and the time elapsed between gel application and sexual intercourse [6]. Nevertheless, the success of the CAPRISA 004 study is documented to be a milestone in the development of an effective vaginal microbicide to prevent the sexual transmission of HIV.

The reduction in HIV infection in women demonstrated by pericoital use of TNF gel in 2010 encouraged the continued development of antiretroviral microbicides. It established that effectiveness depends on consistent and timely use; therefore, design of formulations that women can routinely use with ease is of paramount importance in microbicide development. As a result, attention has shifted toward development of alternative formulations that require less commitment from the user to show efficacy, such as sustained release drug formulations [7,8]. While a broad range of factors contribute to the vulnerability of young women in SSA, a new generation of “women initiated technologies” has the potential to shift dynamic and give women more varied options to manage their own sexual health. As adherence to treatment has been proven as a crucial factor, sustained-release formulations of antiretrovirals may be a viable solution. Promising strategies such as the vaginal ring microbicide formulation may improve adherence and factors that influence it. Vaginal rings have been used to provide sustained and controlled release of various medications including contraception and estrogen replacement, and thus the extension to a sustained-release antiretroviral-containing microbicide for HIV prevention is built upon an already-tested technology [2].

The development of an effective microbicide for HIV prevention involves the selection of potent drugs that can interfere with HIV replication and deliver the drug to the site of replication at the appropriate time. Thus, both the drug and the drug-delivery system are crucial components of a successful product. Since consistent and correct use of the products are critical to their effectiveness, the active pharmaceutical ingredients must be delivered in acceptable vaginal dosage forms. The physical and chemical properties of antiretroviral-based compounds allow for their delivery in formulations designed for flexible dosing. The monthly DPV-VR is one such technology [7,8]. Previous microbicide trials have revealed that women are concerned about communication and consensus about HIV prevention in their relationships, the impact of the prevention method on their partners´ sexual pleasure, and the fear of discovery especially during sex. Thus, using something that would negatively impact male sexual pleasure can also negatively impact intimate partner relationships, increasing hesitancy to take up such control measures by women. Gender dynamics and expectations for sexual relationships and experiences are vital aspects of the context within which sex occurs and, as such, for understanding women´s experience of the ring. Although in SSA where young women are at very high risk of contracting HIV, there is increasing exposure to and emphasis on “modern” powerful and independent women, the predominant feminine identities are still associated with traditional female roles that accept male authority and comply with requests and desires of male partners. This patriarchal influence extends to male decision-making in regards to sexual activity and methods to prevent HIV.

Multiple clinical trials have evaluated the acceptability of the DPV-VR among reproductive-age women in Africa. In these trials, women reported initial fears about the ring´s unfamiliar appearance, placement *in-situ* and side effects, as well as concerns about the ring interfering with sex and/or being noticed by the sexual partner. However, with use and increased familiarity, participants are reported to have appreciated the ring, found it easy to use and integrate into their lives and favored it over other delivery forms such as daily oral pills, condoms or gels. Interventions that include counseling and social support to improve adherence and retention have been carried out. Additionally, the dynamics of participants´ relationships with their male partners have been reported as the most consistently described drivers of ring acceptability and use, with consideration of male partners´ attitudes towards the ring identified as a theme across participant narratives [9]. The initial qualitative analysis and the body of research in this area supports the concept that male partners play an influential role on women´s decision-making around HIV prevention method use, and that the dynamics of a woman´s relationship with her male partner are an important modifier of his influence on her prevention behaviors. For HIV-1 prevention, an antiretroviral-containing vaginal ring could provide long-acting HIV-1 protection while reducing systemic exposure to the active pharmaceutical ingredient and delivering the anti-HIV-1 agent at the site of viral transmission.

Two recent randomised, double-blind, placebo-controlled, phase 3 clinical trials have shown that a 25 mg DPV-VR ring used for 28 days is well tolerated with a favorable safety profile and demonstrated a modest but statistically significant reduction of approximately 30% in HIV acquisition by women [4,7,8]. The ring is made of a flexible silicone matrix polymer and contains DPV that has activity against a broad range of HIV-1 subtypes. It provides sustained release of DPV over a longer period, thereby presenting women with an alternative to daily dosage forms, such as gels. The trials have reported that the protective effect of the DPV-VR ring as compared with placebo, is significant but not as high as hypothesized in the design of the trial. A poor adherence measured by ring residual amounts of DPV has been claimed as one of the main contributor of these results. Non-adherence practices reported include removing the ring briefly for sex, bathing, menses, or multi-week removals with reinsertion one to three days before the clinic visit. Additional reasons reported are related to hygiene concerns, external-influence from peers and family members, and sole interest in study benefits. Also, others may have removed the ring because they wanted to get pregnant or use other vaginal products to prepare for sex [7,8]. Overall, the studies have reported the observation of greater HIV-1 protection among subgroups of women who had evidence of higher rates of adherence in comparison to those with lower rates of adherence. Notably, HIV-1 protection was not observed for women between the ages of 18 and 21 years, and objective markers of adherence were lower in this subgroup than in those older than 21 years. Specifically, HIV protection exceeded 50% in women older than 21 years, who exhibited better adherence than women aged 18-21. Additionally, more detailed analyses using residual levels in rings that had been distributed and returned each month associated the highest levels of ring use with HIV protection, on the order of 75-92%. Among those acquiring HIV, there was no evidence to suggest selection of antiretroviral resistance by DPV exposure.

The creation of an effective microbicide also requires understanding the dynamics of the target population, and transmission prevention strategies must be adapted accordingly. Much of the current effort focuses on understanding the aspects that govern the effectiveness of microbicides, and adherence is a key consideration in the trials. Improving adherence ranges by supporting the users, assessing their perception of risk and analyzing their social background and relationship challenges, including whether their partners allow them to use microbicides. Among the key factors women consider when choosing a vaginally inserted product, whether for HIV prevention or other purposes, is its impact on sexual experiences for themselves and their partners. Ease of use, potential for longer acting protection and discreet use independent of coitus have made rings a focus for development of HIV prevention intervention delivery. It is noteworthy that as with any other interventions in existence, the effectiveness of rings as prevention products is positively correlated with adherence, which is likely to depend on user acceptability of the product. As mentioned earlier, the DPV-VR containing 25 mg DPV was found to be a safe and effective female-controlled method of HIV prevention [4,7,8], marking a major milestone that brings the first long-acting and woman-controlled product a step closer to reaching women.

One of its potential advantages over coitally dependent or daily use products is that it is longer-acting and just requires the user to leave the product in place over the course of the month to be adherent. Following the success of the DPV-VR, two open-label expanded trials among women who were HIV-1-negative who had previously participated in two phase 3 trials, have shown that use of the DPV-VR under conditions approaching real-world scenarios such as visits once every 3 months and less frequent HIV testing is acceptable and results in a reduction in the risk of HIV-1 seroconversion [7]. The findings provide support for improved adherence to the ring once women are aware of the efficacy and safety of the ring and support the feasibility of DPV use to reduce the risk of HIV-1 infection in women, particularly in SSA. Consistent use in an open-label setting, with good tolerability of the DPV-VR, suggest that an HIV-1 prevention method with modest efficacy in which a new ring is used each month, is acceptable and feasible for women in SSA. This implies that the DPV-VR, the first long-acting biomedical method, is a possible HIV-1 prevention option for women.

Though most microbicide research has been undertaken in SSA and Southeast Asia, an effective microbicide will undoubtedly be used worldwide to prevent the sexual acquisition and/or transmission of HIV. Given social, demographic and migratory trends, the population at risk for STIs will continue to grow dramatically. The burden is greatest in SSA, but industrialized nations can also be expected to experience an increased burden of disease because of the prevalence of non-curable viral infections, trends in sexual behavior and increased travel. Based on a systematic review and meta-analysis of available scientific evidence on the long acting, sustained-release anti-HIV microbicidal product, the benefits outweigh the harms. This evidence includes the cost-effectiveness of the DPV-VR, acceptability, demonstrated feasibility, and the potential to increase equity as an additional prevention choice. Limitations of the data include the observed variability in effectiveness in younger age groups that need confirmation in further trials, and the limited data regarding the use of DPV-VR among pregnant and breastfeeding women.

In conclusion, the HIV epidemic not only affects the health of individuals, it also impacts households, communities, and the development and economic growth of nations. With inequalities, power imbalances, violence, marginalization, taboos, stigma and discrimination in LMIC, particularly in SSA, HIV takes hold, and the war against HIV is yet to be worn in some settings in this region [3]. Following the World Health Organisation (WHO) recommendation DPV-VR may be offered as an additional prevention choice for women at substantial risk of HIV infection as part of combination prevention approaches, it should be widely introduced across SSA. This implies that the monthly DPV-VR could help fill a gap by offering women the autonomous use of a long-acting HIV prevention product that they can control and use discreetly to reduce the risk of HIV transmission during vaginal sex. Hence, rolling-out and expanding HIV prevention options by including DPV-VR may not only simplify the use of antiretroviral medications and provide HIV-1 protection to those at high risk, but could also help meet women´s diverse individual needs which are critical in controlling the epidemic and ensuring their sexual and reproductive health and rights. Additionally, the delivery of DPV-VR if layered onto SSA national antiretroviral treatment models, using their supply chains, delivery channels and monitoring systems will expand the HIV prevention method mix and reduce HIV acquisition.
